# An unmet clinical need: roads to remyelination in MS

**DOI:** 10.1186/s42466-019-0026-0

**Published:** 2019-07-08

**Authors:** Peter Göttle, Moritz Förster, Vivien Weyers, Patrick Küry, Konrad Rejdak, Hans-Peter Hartung, David Kremer

**Affiliations:** 1 Department of Neurology, Medical Faculty, Heinrich-Heine-University, Moorenstrasse 5, 40225 Düsseldorf, Germany; 2 Department of Neurology, Medical University of Lublin, Lublin, Poland

**Keywords:** Remyelination, Myelin, Neurodegeneration, Oligodendrocyte, Therapy, Multiple sclerosis

## Abstract

**Background:**

In the central nervous system (CNS) myelin sheaths stabilize, protect, and electrically insulate axons. However, in demyelinating autoimmune CNS diseases such as multiple sclerosis (MS) these sheaths are destroyed which ultimately leads to neurodegeneration. The currently available immunomodulatory drugs for MS effectively control the (auto)inflammatory facets of the disease but are unable to regenerate myelin by stimulating remyelination via oligodendroglial precursor cells (OPCs). Accordingly, there is broad consensus that the implementation of new regenerative approaches constitutes the prime goal for future MS pharmacotherapy.

**Main text:**

Of note, recent years have seen several promising clinical studies investigating the potential of substances and monoclonal antibodies such as, for instance, clemastine, opicinumab, biotin, simvastatin, quetiapin and anti-GNbAC1. However, beyond these agents which have often been re-purposed from other medical indications there is a multitude of further molecules influencing OPC homeostasis. Here, we therefore discuss these possibly beneficial regulators of OPC differentiation and assess their potential as new pharmacological targets for myelin repair in MS.

**Conclusion:**

Remyelination remains the most important therapeutic treatment goal in MS in order to improve clinical deficits and to avert neurodegeneration. The promising molecules presented in this review have the potential to promote remyelination and therefore warrant further translational and clinical research.

## Introduction

**Fig. 1:**
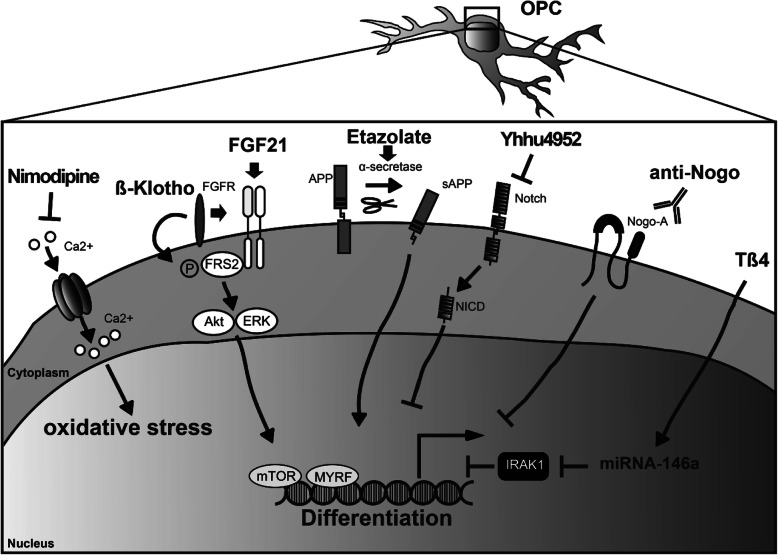
Molecules affecting OPC differentiation. These molecules may represent new therapeutic strategies to exogenously promote remyelination in MS. (s)APP = (secreted ectodomain fragment of APP) ß-amyloid precursor protein; Akt = serine/threonine-specific protein kinase protein kinase B; Ca2+ = Calcium; FGF21 = fibroblast growth factor; FGFR = fibroblast growth factor receptor; FRS2 = fibroblast growth factor receptor substrate 2; IRAK1 = interleukin-1 receptor-associated kinase 1; mTOR = mammalian target of rampamycin; MYRF = myelin regulatory factor; NICD = Notch intracellular domain; OPC = oligodendroglial precursor cell; Tß4 = Thymosin beta-4

Axonal myelin sheaths enable saltatory signal transduction which accelerates information processing 20–100-fold. However, many diseases of the central nervous system (CNS) such as multiple sclerosis (MS) harm or destroy myelin sheaths and the myelin-producing oligodendrocytes resulting in demyelination. MS is an autoimmune inflammatory CNS disease of yet unclear etiology [75, 91]. Its most common clinical course is the relapsing subtype (RMS) which can manifest itself in a plethora of acute clinical symptoms (i.e. relapses) ranging from paresthesias to ataxia or even motor weakness. Most RMS cases ultimately transform into (secondary) progressive MS (PMS) where neurodegeneration outweighs inflammation. Even though there is evidence that adult oligodendrocyte can contribute to myelin repair in the adult CNS [18, 103], remyelination is thought to be mostly mediated by an ubiquitous pool of so-called oligodendroglial precursor cells (OPCs) which can differentiate into mature cells and then generate new myelin [10]. Unfortunately, this spontaneously occurring process called remyelination is overall inefficient and results in shorter and thinner myelin sheaths. Neuropathologically, this is mirrored by the presence of so-called shadow plaques - lightly remyelinated lesions with intermediate levels of myelin. Critical steps for remyelination such as OPC activation, recruitment, differentiation, and ultimately myelin regeneration are orchestrated by a number of extrinsic and intrinsic factors that act either as inhibitors or stimulators of OPC differentiation [47, 50]. Of note, recent publications point to an increasing heterogeneity within the oligodendroglial lineage reflecting differences between white and grey matter localisation and origin [94], different CNS regions [59] as well as lineage alterations upon demyelination [20, 40]. Moreover, as mentioned above, in the diseased adult CNS an additional contribution to myelin repair from partially lesioned oligodendrocytes has recently been suggested [18, 103]. Lastly, the subventricular zone (SVZ) stem cell niche represents yet another source for adult oligodendrogenesis, which is therefore contributing to glial heterogeneity [2]. This review will discuss recently identified molecules affecting OPC differentiation which may represent new therapeutic strategies to exogenously promote remyelination in MS (see Table 1 and Fig. 1 for a respective overview).

**Table 1:** Overview of molecules for potential therapeutic use in MS

Molecule	Mode of action	Effect	Experimental evidence	BBB penetration	Clinical use
Klotho (membrane-bound protein)	Co-receptor of FGFR responsible for FGF23 signaling	Regulation of PTH and vitamin D homeostasis	Immunoprecipitation	No data available	–
Cleaved Klotho (ectodomain of the Klotho protein)	Modulation of the Wnt and insulin/IGF1 pathways Phosphorylation of FRS2, ERK and Akt Activation of mTOR	Promotion of OPC maturation Acceleration of remyelination	Cuprizone-mediated demyelination animal model Knock-out animal model	–	–
Smo (membrane-bound protein)	Activation of the Shh pathway	Acceleration of remyelination	Primary oligodendrocyte cell culture	No data available	–
Clobetasol (synthetic glucocorticoid)	Smo agonist	Acceleration of remyelination	Cerebellar cultures MOG_35–55_ chronic progressive EAE animal model PLP_139–151_ relapsing remitting EAE animal model NMO animal model	+	Treatment of skin disorders (e.g. eczema, psoriasis)
MYRF (transcription factor)	Activation of myelin gene promotors	Promotion of OPC maturation Acceleration of remyelination	Knock-out animal model Lysolecithin animal model Human tissue analysis	No data available	–
Anti-RGMa (antibody)	Neutralization of the proinflammatory and anti-regenerative molecule RGMa Reduction of T cell proliferation Reduction of proinflammatory cytokine secretion	Clinical improvement of EAE Inhibition of inflammation Promotion of neuroregeneration Reduction of proinflammatory microglia	MOG_35–55_ chronic progressive EAE animal model PLP_139–151_ relapsing remitting EAE animal model NOD secondary progressive EAE animal model NMO animal model Human tissue analysis	No data available	Phase 2 clinical trials assessing the anti-RGMa-antibody elezanumab in patients with relapsing (NCT03737851) and progressive MS (NCT03737812)
miR146a (endogenous microRNA)	Inactivation of IRAK1	Promotion of OPC maturation Acceleration of remyelination	Cuprizone-mediated demyelination animal model	No data available	–
AS-2P (ascorbic acid derivate)	Co-factor of hypoxia-inducible factor (HIF) Mediation of ubiquitination and proteasomal degradation of hypoxia-inducible factor α (HIF-α) Antioxidant properties	Promotion of OPC maturation	Mouse neural progenitor-derived OPC cell culture Mouse OPC-dorsal root ganglion neuron coculture Cuprizone-mediated demyelination animal model	No data available (No penetration of ascorbic acid. Only oxidized form can be transported by GLUT1)	Use in the cosmetic industry as AS-2P salts
Tβ4 (hormone-like peptide)	Upregulation of p38MAPK and ILK Anti-inflammatory and immunomodulatory properties	Promotion of OPC proliferation and differentiation Acceleration of remyelination	Immortalized murine N20.1 oligodendrocyte cell culture PLP_139–151_ relapsing remitting EAE animal model Cuprizone-mediated demyelination animal model Human tissue analysis	(+)	Several phase 1 and phase 2 clinical trials assessing the use in patients with dry eye syndrome (NCT02974907, NCT01393132), ulcers (NCT00382174), epidermolysis bullosa (NCT00311766), myocardial infarction (NCT01311518)
Etazolate (pyrazolopyridine derivative)	Alpha-secretase-induced release of the neuroprotective soluble N-terminal APP fragment (sAPPalpha) GABAA receptor modulator Alpha-secretase activator Adenosine antagonist	Promotion of OPC maturation Acceleration of remyelination Protection of myelinated axons from demyelination	Cuprizone animal model Ex vivo lysolecithin-induced demyelination model using cerebellar slices	(+)	Phase 2 clinical trial assessing use in patients with Alzheimer’s disease (NCT00880412)
Nimodipine (calcium channel blocker)	Inhibition of carnitine palmitoyltransferase 1A (Cpt1a) and NADPH oxidase 4 (Nox4) Reduction of reactive nitrogen and oxygen species Induction of microglial apoptosis	Acceleration of remyelination	Mouse primary and N9 microglia cell culture MOG_35–55_ chronic progressive EAE animal model PLP_139–151_ relapsing remitting EAE animal model MP4-induced EAE animal model	+	Treatment of vasospasm following subarachnoid hemorrhage
Hesperidin (flavonoid)	Reduction of proinflammatory cell infiltration into the CNS T-cell polarization of proinflammatory CD4+ T-cells to a regulatory T cell status Antioxidant properties	Reduction of inflammation and demyelination	MOG_35–55_ chronic progressive EAE animal model	+	OTC dietary supplement
Hesperetin (flavonoid)	Downregulation of TLR4 Antioxidant properties	Reduction of inflammation and demyelination	HT22 neuronal and BV-2 microglial cell culture Aβ mouse model Lysolecithin-induced demyelination animal model	+	OTC dietary supplement
Quercetin (flavonoid)	Modulation of the Wnt pathway Release of neurotrophic factors Attenuation of glutamate-mediated excitotoxicity Inhibition of NF-κB and reduction of reactive nitrogen species	Reduction of inflammation and demyelination Acceleration of remyelination Stimulation of neurite outgrowth	Parental PC12 pheochromozytoma and BV-2 microglial cell culture Behavioral and neurochemical studies in swiss albino mice Intracerebral hemorrhage rat model Ethidium bromide-mediated demyelination animal model Lysolecithin-induced demyelination animal model	+	OTC dietary supplement
TDP6 (peptide)	Mimetic of the neurotrophin BDNF Activation of TrkB receptors and downstream Erk1/2	Promotion of OPC differentiation Acceleration of remyelination	Chick and rat primary dorsal root ganglion neuron cell cultures Primary OPC culture Mouse/rat OPC-dorsal root ganglion neuron coculture Cuprizone-mediated demyelination animal model	No data available	–
Anti-NogoA (antibody)	Neutralization of the axonal inhibitory protein Nogo A	Promotion of OPC proliferation and differentiation Acceleration of remyelination Axonal regeneration	Rat, mouse and monkey spinal cord injury animal models Rat stroke animal model MOG_35–55_ chronic progressive EAE animal model Lysolecithin-induced demyelination animal model	No data available*	Phase 1 clinical trials assessing the use of anti-NogoA-antibody ozanezumab in patients with MS (NCT01435993, NCT01424423). Phase 1 clinical trial assessing the use of anti-NogoA-antibody ATI355 in patients with spinal cord injury (NCT00406016). Phase 1 and phase 2 clinical trial assessing the use anti-NogoA-antibody ozanezumab in amyotrophic lateral sclerosis (NCT00875446, NCT01753076).
Yhhu4952 (quinazoline derivate)	Inhibition of the Jagged1-Notch1 pathway	Promotion of OPC proliferation and differentiation Acceleration of remyelination	Rat primary OPC-astrocyte coculture MOG_35–55_ chronic progressive EAE animal model Cuprizone-mediated demyelination animal model	+	–
Tamoxifen (estrogen receptor modulator)	Modulation of estrogen receptors ERα, ERβ, and GPR30	Promotion of OPC differentiation Acceleration of remyelination	Primary OPC culture Ethidium bromide-mediated demyelination animal model	+	Breast cancer treatment
CDP-choline (choline metabolite)	Possible interaction with members of the ERK/MAPK family	Promotion of OPC proliferation Acceleration of remyelination	Primary OPC, microglial and macrophage cell culture MOG_35–55_ chronic progressive EAE animal model Cuprizone-mediated demyelination animal model	+	OTC dietary supplement Several clinical trials assessing the use in patients with acute stroke (e.g. NCT00331890), traumatic brain injury (e.g. NCT00545662) or psychiatric disorders (e.g. NCT00223236) among others.

*BBB* Blood brain barrier, *MS* Multiple sclerosis, *OPC* Oligodendroglial precursor cell, *PTH* Parathyroid hormone, *MOG* Myelin oligodendrocyte glycoprotein, *PLP* Proteolipid protein, *EAE* Experimental autoimmune encephalomyelitis, *NMO* Neuromyelitis optica, *MP4* MBP-PLP fusion protein, *NOD* Non-obese diabetes, *OTC* Over the counter, + = penetrates the blood brain barrier, (+) = only little evidence for BBB penetration or low permeability, − = does not penetrate the blood brain barrier, * = Anti-NogoA antibody was administered intravenously in clinical trials with MS patients, but pharmacokinetic data was not publicly accessible

### Receptors/membrane-bound molecules

#### Klotho

As a single-pass transmembrane protein, Klotho is primarily found in the kidney and the CNS [52]. As a result of ectodomain shedding, cleaved Klotho can act as a ligand, hormone and/or glycosidase regulating ion homeostasis. Furthermore, it modulates the Wnt and insulin/IGF1 pathways which represses IGF-1 signaling [51]. Recent studies found that Klotho accelerates remyelination in cuprizone-mediated demyelination animal models [92]. In this context, it is important to note that by means of a knock-out animal model Klotho had previously already been shown to promote OPC maturation via the phosphorylation of FRS2, ERK and Akt leading to an activation of mammalian target of rapamycin (mTOR; [12]). However, given its inability to cross the blood-brain barrier (BBB), a small molecule approach aimed at increasing endogenous Klotho production appears to be the most promising therapeutic strategy [1]. Moreover, the uncleaved Klotho transmembrane protein acts as a co-receptor forming a complex with fibroblast growth factor receptor (FGFR) responsible for FGF23 signaling which is involved in vitamin D production [72, 73]. Regarding other members of the FGF family, β-klotho was demonstrated to enhance remyelination in a toxic demyelination animal model based on an interaction with FGF21. This effect seems to result from a stimulation of OPC proliferation promoting CNS regeneration [53].

#### Smoothened (Smo)

Besides the already mentioned Wnt, insulin/IGF and FGF signaling pathways, sonic hedgehog (Shh) signaling seems to play an important role in OPC differentiation. Inhibition of the atypical G protein-coupled receptor Smoothened (Smo), an activator of Shh signaling, caused a dramatic decrease in myelin marker expression such as myelin basic protein (MBP) and myelin associated glycoprotein (MAG) during OPC differentiation in cell culture [95]. As a result, Smo constitutes a potential therapeutic target for myelin repair [14]. In vitro cell culture-based drug screening approaches and functional remyelination studies (both in ex vivo cerebellar cultures and a neuromyelitis optica animal model) identified a number of active compounds with remyelination/myelin repair properties that act as Smo agonists, such as, for instance, clobetasol which is a corticosteroid already approved for the treatment of skin diseases such as psoriasis (CLOB; [65, 71, 102]).

#### Myelin regulatory factor (MYRF)

Myelin regulatory factor (MYRF) is a membrane-bound transcription factor tightly associated with the endoplasmic reticulum (ER; [19, 34]). As revealed by MRF conditional knockout (CKO) mice, upon self-cleavage, the N-terminal fragment of MYRF translocates from the ER into the nucleus where it functions as a transcription factor stimulating myelin gene promotor activity [19]. Hence, it seems to be critical for oligodendroglial differentiation and myelin maintenance [46]. Studies in lysolecithin-induced demyelination animal models point to an essential role of MYRF in remyelination/myelin repair which is underlined by the observation that in chronic human MS lesions oligodendrocytes were found to lack MYRF expression [17].

#### Repulsive guidance molecule a (RGMa)

Repulsive guidance molecules (RGMs) are a small family of membrane-bound proteins with tissue-specific expression which were initially described as axonal guiding factors during embryogenesis. However, recently they were also recognized as being involved in multiple cellular processes such as axon guidance during adulthood, neuronal survival, axonal regeneration, iron metabolism and skeletal development [83]. As a member of this family, RGMa was identified as a regulatory factor potentially facilitating inflammation and inhibiting regeneration and remyelination in MS [15]. RGMa acts mainly through its target receptor neogenin which it activates differently depending on its previous proteolytic processing: either involving activation of LARG, RhoA, and ROCK or via γ-secretase cleavage of the intracellular domain of neogenin [4]. In CD4+ T cells, RGMa leads to an activation of the GTPase Rap1 resulting in an increased adhesion of T cells to intracellular adhesion molecule-1 (ICAM-1; [63]). However, although not directly inhibiting T cell trafficking to the CNS, the use of murine EAE (experimental autoimmune encephalomyelitis) animal models demonstrated that treatment with anti-RGMa antibodies resulted in a diminished T cell proliferation as well as inflammation as indicated by a reduced IL-2, IL-4, IFN-γ and IL-17 secretion. Accordingly, the clinical course of EAE was improved as compared to controls [63]. Of note, this beneficial therapeutic effect was also confirmed in an SPMS animal model, where anti-RGMa antibody treatment prevented secondary progression of EAE, inhibited inflammation and promoted neuroregeneration in the murine spinal cord leading to functional recovery [89]. In addition, this study showed that anti-RGMa antibody treatment reduced the number and activation of CD11b + microglia in the inflamed CNS. Interestingly, activated microglia were also shown to express RGMa and to inhibit axonal outgrowth via direct cell to cell contact. This effect could be reversed not only by the anti-RGMa antibody but also by the antibiotic minocycline recently tested in clinical trials as a potential treatment option for clinically isolated syndrome (CIS) [45, 61]. In addition, RGMa may also be involved in neuromyelitis optica (NMO) as in an NMO animal model similar effects of anti-RGMa treatment were observed such as a more favourable disease course, a weaker immune response, and a partial restoration of AQP4 and GFAP reactivity [33].

### Physiologically occurring free molecules

#### miR-146a

During the past years, microRNAs and their dysregulated expression have been studied extensively in MS pathology [16]. Pharmaceutically, they are of interest due to the idea that they could be delivered to target structures via extracellular vesicles [69]. MicroRNA miR-146a was assigned a potential role in myelin repair as it was demonstrated to promote oligodendroglial differentiation and to enhance remyelination in the toxically demyelinated corpus callosum of mice [108]. Mechanistically, it is thought to inactivate interleukin-1 receptor-associated kinase 1 (IRAK1), an intracellular signaling molecule disturbing OPC differentiation [48].

#### L-ascorbyl-2-phosphate (AS-2P)

Various studies have demonstrated decreased levels of ascorbic acid in the serum of MS patients [7, 90]. In this context, in vitro and in vivo experiments recently identified L-ascorbyl-2-phosphate (AS-2P), a stable form of vitamin C, to be able to stimulate OPC differentiation into mature and myelinating oligodendrocytes [31]. AS-2P was found to increase the expression of oligodendroglial myelin markers such as 2′,3′-Cyclic-nucleotide 3′-phosphodiesterase (CNPase) and MBP and to facilitate the formation of myelin sheaths in OPC/neuron co-cultures. In addition, As-2P also exerts a regenerative impact in the toxic cuprizone animal model. Interestingly and of note, all these effects seem to be independent of its well-described antioxidant properties.

#### Thymosin-β4

Thymosin beta-4 (timbetasin, Tβ4) is a 43-amino acid hormone-like peptide which is mainly involved in the regulation of cell motility and migration. It is thought to interact with globular actin (G-actin) regulating actin polymerization and ultimately microfilament formation [78]. Furthermore, recent studies demonstrated Tβ4 to have anti-inflammatory and immunomodulatory properties, to promote OPC proliferation and differentiation as well as remyelination in the CNS. In EAE and the cuprizone model, Tβ4 treatment led to a significant neurological functional improvement which was accompanied by a reduction of inflammatory cell infiltrates, an enhanced oligodendrogenesis and recruitment of OPCs to demyelinated axons which resulted in a relevant reduction of axonal damage in the demyelinating CNS [107, 109]. In MS brains, it was found to be present in the periphery of not yet fully remyelinated lesions which, in the context of the previous findings, suggests a restorative effect [57]. On the other hand, proteomic profiling studies of cerebrospinal fluid (CSF) showed significantly decreased Tβ4 levels in MS patients as compared to patients with other neurological diseases [55]. Mechanistically, the exact mode of action of Tβ4 still needs further clarification but seems to be multifaceted as Tβ4 has several biological functions [25]. However, Tβ4 leads to an upregulation of p38 mitogen-activated protein kinase (p38MAPK; [79]), miRNA 146a [80, 108, 111], and smoothened-activating integrin linked kinase (ILK) [44], all of them being involved in the mediation of OPC differentiation (see further above).

### Non-physiologically occurring free molecules

#### Etazolate

The pyrazolopyrine derivate etazolate features unique pharmacological properties as it simultaneously acts as an alpha-secretase activator, an allosteric GABAA receptor modulator [58], an adenosine antagonist [97] and a phosphodiesterase (PDE) inhibitor for PDE4 [96]. In demyelinating animal models etazolate has been shown to promote the restoration of myelinated axons via an alpha-secretase-induced release of the soluble N-terminal APP fragment (sAPPalpha), an endogenous protein with neuroprotective properties [56]. Of note, a clinical trial in Alzheimer’s disease (AD) already demonstrated a favorable clinical safety/tolerability profile (ClinicalTrials.gov Identifier: NCT00880412).

#### Nimodipine

The voltage gated L-type calcium channel blocker nimodipine is commonly used clinically to prevent cerebral vasospams often following subarachnoid haemorrhage (SAH). In different EAE animal models and in cell culture experiments nimodipine was demonstrated to exert a positive impact on both inflammation and neurodegeneration via the induction of microglial apoptosis and improved oligodendrogenesis [81]. Of note, it does so without affecting the primary immune response. As it exerts a protective effect on neurons and is able to preserve myelin in EAE, nimodipine seems to be a promising candidate for both RMS and PMS [39].

#### Hesperidin and hesperetin

Hesperidin is a common flavanone from the fruit peel of *Citrus aurantium* (bitter orange) with antioxidant, anti-inflammatory and neuroprotective properties [77]. Oxidative stress is assumed to be one of the causes of neurodegeneration in MS [26] so that antioxidant compounds have long been regarded as a protective therapy option [27, 35, 84]. In MOG-induced EAE hesperidin treatment led to a reduction of disease severity. This effect is probably mediated by a hesperidin-induced reduction of proinflammatory cell infiltration into the CNS, a T-cell polarization of proinflammatory CD4+ T-cells to a regulatory T cell status and a subsequent shift from proinflammatory IL-1b, IL-6, IL-17, and TNF-α to regulatory L-10 and TGF-β cytokines [13, 32]. In addition, the aglycone derivate of hesperidin (hesperetin) inhibits neuroinflammation by a downregulation of TLR4 which leads to a decrease of the proinflammatory TLR4-dependent downstream cytokines NF-κB, TNF-α, and IL-1β as demonstrated in an Aβ mouse model [36]. The administration of hesperetin in a lysolecithin (LPC)-induced focal demyelination animal model demonstrated a decreased demyelination and glial activation in the optic chiasm which led to a functional recovery and might reflect a beneficial impact of hesperetin on remyelination [5].

#### Quercetin

Like hesperidin, the flavonol quercetin is a member of the flavonoid group and thus considered to possess antioxidant, antiviral and neuroprotective properties. Its neuroprotective effect is mainly based on its anti-inflammatory and antioxidant activity as well as its ability to induce the release of neurotrophic factors while simultaneously attenuating glutamate-mediated excitotoxicity [88]. Quercetin treatment was shown to reduce the expression levels of the pro-inflammatory cytokines TNF-α, IL-6, IL-1β and COX-2 [60] promoting a functional recovery in the inflamed CNS based on an animal model of intracerebral hemorrhage [110]. Cell culture experiments revealed that quercetin enhances neurite outgrowth by the release of neurotrophic factors, such as brain-derived neurotrophic factor (BDNF), neurotrophin (NGF; [66]), growth-associated protein 43 (GAP43) and microtubule-associated protein (MAP; [62]). Furthermore, it can potentially decrease nitrosative stress by inhibiting NF-κB and its downstream effector inducible nitric oxide synthase (iNOS; [43]). Regarding remyelination, recent studies demonstrated that quercetin interferes with the canonical Wnt signaling pathway by separating the Wnt-downstream transcription regulating protein β-catenin from transcription factor 4 [21, 22, 104], which in turn improves myelin repair as demonstrated in demyelinating animal models [8, 64].

#### Tricyclic dimeric peptide 6 (TDP6) – BDNF

Tricyclic dimeric peptide 6 (TDP6) is a small multicyclic peptide that mimics a distinct region of the neurotrophin BDNF which exerts its effect via the transmembrane tropomyosin-related kinase B (TrkB) receptor [11]. BDNF is required for normal CNS myelination [9, 41] and is able to directly enhance myelination in oligodendrocytes [100]. Cell culture studies demonstrated, that TDP6 selectively targets TrkB receptors [67] and activates the downstream signaling molecules extracellular related-kinase 1 and 2 (Erk1/2; [98]) which in turn promotes remyelination [24, 99]. Further studies in EAE demonstrated that stimulation of TrkB enhances remyelination by increasing oligodendrocyte differentiation, the frequency of myelinated axons and myelin sheath thickness [23].

#### Miscellaneous other molecules

Anti-NogoA antibodies were previously developed for and intensively studied in axonal regeneration of the acutely injured CNS [82]. Their application in a rodent lysolecithin-based demyelinating animal model was found to promote remyelination of axon tracts in the spinal cord [37]. This indicates that anti-NogoA treatment might constitute a potential anti-degenerative approach particularly interesting regarding PMS in which axonal degeneration is thought to outweigh inflammation [38]. Aside from that, Notch signaling has been found to play an important role in the regulation of developmental and repair-associated oligodendrogenesis [42]. Interestingly, it was now shown that the small molecule Yhhu4952 can be used to inhibit the Jagged1-Notch1 pathway and when applied to cuprizone-demyelinated or EAE animal models it was found to boost oligodendroglial differentiation and to improve myelin restoration [106]. Besides these factors tamoxifen, an FDA-approved selective estrogen receptor modulator (SERM) used for the treatment of breast cancer, was found to induce OPC differentiation in culture and to accelerate remyelination in demyelinating animal models [28]. Further studies demonstrated that modulation of estrogen receptors ERα, ERβ, and GPR30 are responsible for the observed effects [6]. Another interesting approach is to use choline metabolites such as CDP-choline (citicoline) to enhance remyelination in MS [29, 30, 76, 85]. CDP-choline was found to ameliorate the disease course of EAE and to exert beneficial effects on myelin, oligodendrocytes and axons. Upon cuprizone-induced demyelination, CDP-choline stimulated myelin regeneration and reversed motor coordination deficits. Mechanistically, increased remyelination apparently arises from an increase in the numbers of proliferating OPCs and oligodendrocytes [86].

## Conclusion

Remyelination remains the most important therapeutic treatment goal in MS in order to improve clinical deficits and to avert neurodegeneration which is already present at early stages of the disease [49]. This is of particular importance as neurodegeneration dictates in large measure the accumulating neurological disability in MS. Even in radiologically isolated syndrome (RIS), a controversially discussed precursor to MS based exclusively on the incidental finding of clinically asymptomatic MRI lesions, there is evidence for thalamic volume loss as a correlate of early neurodegeneration [3]. It is therefore very encouraging to observe the plethora of emerging clinical studies investigating the potential of different agents to promote remyelination. Once fully established, such therapies will probably be used as add-on medications in a two-pronged approach alongside „classical “immunomodulators. Of course, translation of experimental findings into clinical trials assessing remyelination faces significant challenges. Notably it is unclear which outcome measures should be best used in order to adequately monitor therapeutic efficiency [68, 87]. Secondly, molecules supposed to stimulate OPC differentiation should not exert negative pleiotropic effects on otherwise physiologically required homeostatic pathways. This is particularly true for experimental molecules aiming at a modulation of the activity of receptors and membrane-bound proteins such as, for instance, Klotho, Wnt, Smo and RGMa. Accordingly, for entirely new molecules extensive animal studies will be an absolute prerequisite as a first line of verification that they are well-tolerated and do not lead to serious adverse events (SAEs). In contrast, repurposing of well-known drugs already approved for other medical indications constitutes an elegant strategy to avoid SAEs, to accelerate clinical development and to secure patient trust. Of note, in order to produce effects on remyelination potential molecules need to be able to cross the BBB into the brain. It is therefore key to find effective ways of delivery, all the more, as it is a misconception that even most so-called small molecules (i.e. < 900 Da) can easily cross the BBB [70]. At least for Clobetasol [65], Yhhu4952 [106], tamoxifen [54], nimodipine [93], CDP-choline [74] and the flavonoids [101, 105] BBB penetration has already been established (see Table 1). Despite all these challenges, looking at the multitude of promising molecules presented in this review is extremely reassuring.

## Data Availability

Data sharing is not applicable to this article as no datasets were generated.

## Abbreviations

AD: Alzheimer’s disease
AS-2P: L-ascorbyl-2-phosphate
BBB: Blood-brain barrier
BDNF: Brain-derived, neurotrophic factor
CIS: Clinically isolated syndrome
CLOB: Clobetasol
CNPase: 2′,3′-Cyclic-nucleotide 3′-phosphodiesterase
CNS: Central nervous system
CSF: Cerebrospinal fluid
EAE: Experimental autoimmune encephalomyelitis
ER: Endoplasmatic reticulum
Erk1/2: Extracellular related-kinase 1 and 2
FGFR: Fibroblast growth factor receptor
G-actin: Globular actin
GAP43: Growth-associated protein 43
ICAM-1: Intracellular adhesion molecule-1
ILK: Integrin linked kinase
iNOS: Inducible nitric oxide synthase
IRAK1: Interleukin-1 receptor-associated kinase
LPC: Lysolecithin
MAG: Myelin associated glycoprotein
MAP: Microtubule-associated protein
MBP: Myelin basic protein
MS: Multiple sclerosis
mTOR: Mammalian target of rapamycin
MYRF: Myelin regulatory factor
NGF: Neurotrophin
NMO: Neuromyelitis optica
OPC: Oligodendroglial precursor cells
p38MAPK: p38 mitogen-activated protein kinase
PDE: Phosphodiesterase
PMS: Progressive multiple sclerosis
RGM: Repulsive guidance molecule
RIS: Radiologically isolated syndrome
RMS: Relapsing multiple sclerosis
SAE: Serious adverse event
SAH: Subarachnoid haemorrhage
sAPPalpha: Soluble N-terminal amyloid precursor protein alpha
SERM: Selective estrogen receptor modulator
Shh: Sonic hedgehog
Smo: G protein-coupled receptor Smoothened
TDP6: Tricyclic dimeric peptide 6
Tf4: Transcription factor 4
TrkB: Tropomyosin-related kinase B
Tβ4: Thymosin beta-4
